# A Robust PCR Protocol for HIV Drug Resistance Testing on Low-Level Viremia Samples

**DOI:** 10.1155/2017/4979252

**Published:** 2017-04-03

**Authors:** Shivani Gupta, Tracy Taylor, Aileen Patterson, Binhua Liang, Jared Bullard, Paul Sandstrom, Gary Van Domselaar, Hezhao Ji

**Affiliations:** ^1^Department of Medical Microbiology, University of Manitoba, Winnipeg, MB, Canada; ^2^The Bioinformatics Core, National Microbiology Laboratory, Public Health Agency of Canada, Winnipeg, MB, Canada; ^3^National HIV & Retrovirology Laboratories, JC Wilt Infectious Diseases Research Center, Public Health Agency of Canada, Winnipeg, MB, Canada; ^4^Department of Biochemistry and Medical Genetics, University of Manitoba, Winnipeg, MB, Canada; ^5^Cadham Provincial Laboratory, Winnipeg, MB, Canada; ^6^Department of Pediatrics and Child Health, University of Manitoba, Winnipeg, MB, Canada

## Abstract

The prevalence of drug resistance (DR) mutations in people with HIV-1 infection, particularly those with low-level viremia (LLV), supports the need to improve the sensitivity of amplification methods for HIV DR genotyping in order to optimize antiretroviral regimen and facilitate HIV-1 DR surveillance and relevant research. Here we report on a fully validated PCR-based protocol that achieves consistent amplification of the protease (PR) and reverse transcriptase (RT) regions of HIV-1* pol* gene across many HIV-1 subtypes from LLV plasma samples. HIV-spiked plasma samples from the External Quality Assurance Program Oversight Laboratory (EQAPOL), covering various HIV-1 subtypes, as well as clinical specimens were used to optimize and validate the protocol. Our results demonstrate that this protocol has a broad HIV-1 subtype coverage and viral load span with high sensitivity and reproducibility. Moreover, the protocol is robust even when plasma sample volumes are limited, the HIV viral load is unknown, and/or the HIV subtype is undetermined. Thus, the protocol is applicable for the initial amplification of the HIV-1 PR and RT genes required for subsequent genotypic DR assays.

## 1. Introduction

Effective antiretroviral therapy (ART) for HIV/AIDS has successfully converted the fatal disease into a manageable chronic infection [1]. The majority of patients adhering to a prescribed ART regimen achieve sustainable viral suppression, as indicated by undetectable plasma viral load (VL) (<50 copies/mL) [2]. However, drug resistance (DR) has become an obstacle in maximizing the clinical benefit of ART, and thus routine genotypic HIV DR testing is recommended for all HIV-infected patients for HIV DR surveillance and/or clinical monitoring purposes. Conventional HIV DR typing relies on sequencing-based analysis of HIV genes encoding ART-targeted viral proteins, primarily HIV protease (PR) and reverse transcriptase (RT), against the reference HIV DR mutation database [3]. Successful PCR amplification of these genes is essential, regardless of which method is used subsequently for HIV genotyping.

PCR amplification of the HIV PR and RT gene region is beset by several problems, namely, the genomic sequence diversity inherent as viral quasispecies within infected patients, as well as across populations, while many HIV clades/subtypes are circulating. For this reason, universal primers and protocols that consistently amplify target HIV-1 genes across various HIV subtypes are invaluable [4, 5]. Another challenge to amplification is the low amount of genomic template available when a patient's VL is low, exampled by residual low-level viremia (LLV). Instead of continued viral suppression while on ART, a small portion of patients may first experience a rapid VL reduction episode but then retain a low but detectable VL level for an extended time period despite continued ART. This condition is referred to as LLV, and it is characterized by VL within 50~1,000 copies/mL for over 6 months while on ART [6]. Although the mechanisms of LLV remain unsolved, accumulating evidence suggests that LLV reflects an incomplete viral suppression and persistent LLV may lead to increased occurrence of HIV drug resistance (DR) [7], potential viral rebound [8, 9], and persistent disease progression [6]. The potential influence of low abundance DR variants (LADRVs) harbored by patients with LLV against prescribed ART regimen reinforces the need for a reliable protocol that amplifies viral RNA from LLV samples for further HIV DR genotyping [10].

Previous publications describing the protocols for the amplification of low copy number HIV-1 templates have suffered from limitations such as low consistency, irreproducibility due to lack of technical details, narrow HIV subtype coverage, and/or limited sensitivity. Moreover, most of these methods require a high plasma input volume of more than 1 mL [10–13]. In the current study, we present a well-optimized PCR protocol that largely overcomes these limitations and is robust, sensitive, and reproducible for different HIV-1 subtypes at varied VL levels.

## 2. Materials and Methods

### 2.1. Specimens and Controls

The specimens applied in the development and validation of this protocol included the following: (1) External Quality Assurance Program Oversight Laboratory (EQAPOL) HIV-spiked plasma samples with varied HIV-1 subtypes at known viral loads; (2) thirty-six LLV plasma samples collected from a Kenyan HIV-infected children cohort; (3) sixteen anonymous HIV+ plasma specimens collected from Canadian adult patients with known viral loads; (4) normal human plasma (NHP) and Accurun samples from SeraCare, USA, as negative and positive controls, respectively. The NHP was also applied for sequential dilution of the EQAPOL specimens to mimic plasma samples at varied VL levels, including those in the LLV range. All relevant reagents were acquired from indicated commercial sources. Scientific and ethical approval for this study was granted by the Research Ethics Board of the University of Manitoba (HS19999 (B2016:078)) and by the Scientific and Ethics Review Unit (KEMRI-SERU) of the Kenya Medical Research Institute (protocol #SSC-2500). Institutional approval for data collection and access to the medical records and plasma samples was granted by the Nyumbani Medical Board (NMB), which oversees the Leo Toto Program (LTP) from which the HIV-1 infected Kenyan children specimens were acquired. The NMB granted the researchers consent on behalf of the caregivers and children. Patient confidentiality was ensured by substituting personally identifiable information with a unique alphanumerical code to each patient in the study.

### 2.2. HIV RNA Extraction and Reverse Transcription PCR (RT-PCR)

The optimized protocol started with the HIV viral RNA isolation from patients' plasma specimens using a spin column-based QIAamp Viral RNA extraction kit (Qiagen) as per the manufacturer's instructions [14]. The initial input volume of plasma specimen was 400 *μ*L and the RNA extracts were recovered using an elution volume of 60 *μ*L. Viral RNA extraction was followed by one-step RT-PCR using Invitrogen Superscript III One-step PCR kit (Invitrogen, Canada) with Platinum High Fidelity Taq (Invitrogen, Canada). The RT-PCR primers are described in Table 1 and the derived amplicon encompasses nucleotides 2,071~3,370 of HIV-1* pol* gene as referring to HIV-1 HXB-2 sequence (GenBank Accession number: K03455) [14, 15]. The 50 *μ*L RT-PCR reaction contains 1x SuperScript III reaction buffer, 1.6 mM MgSO_4_, forward and reverse primers at 200 nM each, 1 *μ*L SuperScript III enzyme mix, and 12 *μ*L of RNA extracts. The RT-PCR was performed on a GeneAmp PCR system 9700 thermocycler (ABI, USA) with cycling conditions as follows: (1) an initial reverse transcription step of 30 min at 50°C; (2) 2 min at 94°C for DNA denaturation and Taq DNA polymerase activation; (3) 40 cycles of 20 s at 94°C for DNA melting, 30 s at 50°C for annealing, and 1.5 min at 68°C for elongation; (4) a final extension step of 5 min at 68°C; and (5) 4°C till the end.

### 2.3. Nested PCR

To further amplify the target genes, Phusion Hot Start II High Fidelity DNA Polymerase kit (Life Technologies, Canada) was used for nested PCR with primers described in Table 1 [14, 15]. The derived amplicon spans nucleotides 2,243~3,326 of HIV-1* pol* gene covering the entire PR and first 258 amino acids of RT genomic regions. The 50 *μ*L PCR reaction contained 1x HF buffer, 200 *μ*M of dNTP, forward and reverse primers at 200 nM each, 1 unit of Phusion enzyme, and 5 *μ*L RT-PCR amplicon. Amplification was carried out with the following cycling conditions: (1) an initial DNA denaturation and enzyme activation step of 30 s at 98°C; (2) 35 cycles of 10 s at 98°C for DNA melting, 20 s at 62°C for annealing, and 40 s at 72°C for elongation; (3) a final extension step of 10 min at 72°C; and (4) 4°C till the end. The derived PCR amplicons were evaluated both qualitatively and quantitatively using QIAxcel (Qiagen, Canada) which is an automated capillary DNA electrophoresis system. Alternatively, they could be visualized simply through agarose gel electrophoresis using GelGreen nucleic acid staining (10,000x in water, Biotium).

### 2.4. Protocol Optimization and Validation

The protocol described above was first established using EQAPOL HIV-spiked plasma samples with known viral loads. These samples primarily covered 4 major HIV-1 subtypes A1, B, C, and D (Table 2). Prior to HIV RNA extraction, serial dilution was performed on all EQAPOL samples to obtain a VL range of 5,000, 2,500, 1,000, 500, 200, 100, and 50 copies/mL to simulate the scenarios of LLV. The consistency, sensitivity, and efficiency of this protocol were further assessed using sequentially diluted clinical specimens at known VL levels from a Canadian cohort. Finally, it was validated using specimens collected from a Kenyan patient cohort with confirmed LLV status and known viral load, but unknown HIV-1 subtypes.

During the development of this protocol, assessments and comparisons of alternative experiment designs were also conducted for different HIV viral RNA extraction approaches (manual extraction with QIAamp Viral RNA extraction kit versus silica bead-based HIV RNA extraction using NucliSens easyMAG system (BioMérieux, Canada)), different RT-PCR protocols (one-step versus two-step RT-PCR), and different nested PCR protocols (one-amplicon approach as described above versus two-amplicon approach which amplifies the HIV-1 PR and RT gene separately) along with various volumes and concentrations of the reagents, templates, or primers used. The details of all primers pertaining to the RT-PCR and both nested PCR options are depicted in Table 1. Notably, well-optimized in-house assays for HIV-1 genotyping have been the primary option for most HIV DR reference laboratories for economical and PCR efficiency reasons. Therefore, commercial assays were not taken into account for comparison while we assessed the performance of this new protocol.

## 3. Results and Discussion

Efficient HIV viral RNA recovery with proper RNA extraction approach is essential in ensuring the success of subsequent molecular assays. The instrumental and technical requirements of different viral RNA extraction protocols vary drastically. Automated NucliSENS easyMag extraction system applies silica bead-based HIV RNA extraction and it has been routinely used in many HIV reference laboratories including ours with proved efficiency. However, it required highly specialized instrument and accessories that are not commonly available especially in laboratories in resource limited setting (RLS). We had assessed the performance of spin column-based manual extraction approach with QIAamp Viral RNA extraction by comparing it with automated NucliSENS easyMag protocol. The manual Qiagen RNA extraction method described above was found to be equally efficient on all EQAPOL control samples with comparable yield (data not shown). We then examined the optimal plasma input and RNA elution volumes at different VL levels which renders downstream PCR success. Our results demonstrated that a combination of 400 *μ*L initial plasma input and 60 *μ*L elution performs reliably for LLV samples while using manual RNA extraction protocol, which is more practical and cost-effective in general laboratories.

The initial RT-PCR step aims to convert HIV viral RNA into complimentary DNA through reverse transcription, and the target viral gene fragments are then amplified. It could be conducted through one-step RT-PCR in which the combined RNA conversion and gene-specific PCR amplification occur in a single tube reaction. In contrast, two-step RT-PCR requires two separate reactions for RNA conversion and gene-specific amplifications, which requires more hands-on time, but it is more flexible as one RT reaction may provide templates for multiple downstream PCR reactions. With minimized human interference and simplified workflow, one-step RT-PCR is preferred when possible. While we compared the two approaches in this study, we observed that one-step RT-PCR performed consistently better, likely attributable to the higher abundance of templates available for the PCR amplification step.

Nested PCR aims to further amplify the target gene fragment and reduce potential contamination resulting from the nonspecific amplification due to unintended primer binding during RT-PCR. Typically, a larger outer fragment is unambiguously amplified with RT-PCR from which a smaller inner fragment is subsequently amplified via nested PCR. The HIV DR associated mutations reside in the HIV PR gene that is 297 nucleotides in length, and the first 690 nucleotides of the HIV RT gene encode the amino acids 1 to 230 of the RT protein. Therefore, the target gene fragment for HIV DR analysis is approximately 1,000 bp in length. The PCR amplification of this region could be carried out with a single reaction (one-amplicon approach) or two reactions (two-amplicon approach). One-amplicon nested PCR was fast and easy requiring only one PCR reaction, which also reduces pipetting errors. In comparison, two-amplicon approach involves two separate reactions and two primer sets amplifying the HIV PR and RT gene targets separately, but the PCR efficiency could be improved theoretically since shorter amplicons are to be generated. This is especially helpful when significant viral RNA degradation may exist. We have compared the performance of the two approaches in PCR amplifying the examined EQAPOL controls as well as clinical specimens. Our results indicated that one-amplicon nested PCR worked consistently on all examined specimens, suggesting that the possible RNA degradation may be less concerning when only a small viral gene fragment (~1,000 bp) is targeted.

By targeting the well-conserved regions in the pol gene and incorporating degenerative bases, the primers applied here for both RT-PCR and nested PCR amplifications accommodate all major HIV-1 subtypes and circulating recombinant forms (CRFs) although only 4 dominant subtypes were examined here [4, 5]. In fact, these PCR protocols also accommodate other HIV-1 subtypes and CRFs, such as subtypes F, G, CRF01_AE, and CRF02_AG, when applied in other studies as LLV is not concerned (data not shown). Both PCR kits were selected for the use of high fidelity polymerases and are from reputable commercial resources with proven performance consistency. It is noteworthy that the success rates of nested PCR amplification are highly dose-dependent, suggesting that the initial HIV-1 template input at the RT-PCR stage and the RT-PCR efficiency determine the eventual PCR outcome. For this protocol, 400 *μ*L of plasma was used for RNA extraction and eluted in 60 *μ*L volume and then 12 *μ*L of RNA extract was introduced into RT-PCR step. With the assumption of 80% extraction efficiency, the HIV RNA input is estimated at ~15 copies per RT-PCR reaction if the initial plasma VL is at 500 copies/mL. Even with such low viral template input, the protocol worked efficiently on subtypes A1 and B even when VL is at 100 copies/mL. In comparison, the sensitivity of this protocol for subtype C is determined at 500 copies/mL (Table 3). Notably, the protocol performed less consistently on HIV-1 subtype D when the VL is ≤1,000 copies/mL and this is observed in several repeated experiments conducted by different technicians (data not shown), suggesting that this is largely due to the nature of samples, but not technical artifacts from the manual sample dilution. Close examination of the intersubtype variations indicated that the unique genetic composition of HIV-1 subtype D at the primer binding sites, especially those for primers RT_3370_R1 and PR_2243_F2, may be responsible for the reduced PCR consistency observed in this study [15]. Considering the limited number of HIV templates available in LLV specimens, this observation may highlight the issue of biased amplification when HIV-1 subtype D specimens are examined in the context of LLV. Less likely, the reduced PCR efficiency in this case may be explained by the reduced primer binding efficiency due to the unique secondary viral RNA/DNA structure in subtype D which could impair the PCR efficiency subsequently.

While being assessed using a cohort of anonymous Canadian clinical specimens with known HIV VLs ranging from 3.83 million to <50 copies/mL, the protocol successfully amplified all samples except for those with VL < 100 copies/mL, further confirming the efficiency of this protocol in amplifying samples with unknown VLs and/or subtypes. Moreover, the protocol was also tested using 36 plasma samples from HIV-1 infected children with confirmed LLV in Nairobi, Kenya. The VLs of these samples ranged from 169 to 926 copies/mL, averaged at 500 copies/mL. It is noteworthy that all these specimens had been previously used in other assays and not properly stored and/or had insufficient volume left. For instance, the initial plasma volume was between 50 *μ*L and 500 *μ*L, to some of which PBS solution had to be added to increase the volume in order to initiate manual viral RNA extraction. Despite the poor sample quality, this protocol performed well, yielding a 63% PCR success rate that is significantly higher than previously reported protocols [13, 16]. Some of these specimens were then successfully sequenced using Illumina MiSeq technology in a relevant study for HIV DR mutation analysis (data not shown), confirming that this protocol satisfies the need of DRM analysis for patients expressing LLV. However, extra consideration must be given to the viral copy number in the PCR reaction when amplicons from LLV samples are subject to sequencing analysis for detecting LADRVs. A low copy number of HIV templates in the PCR reaction does not allow for reliable identification and quantification of LADRVs. For instance, if the patient VL is 50 copies/mL, the initial HIV RNA template introduced into the RT-PCR step is ~1.5 copies and all progeny amplicons are derived from them. One should not expect to reliably detect any DR variants present under 50% relative abundance with this limited input template number. The protocol described here enables reliable PCR amplification of LLV HIV-1 specimens with various subtypes. However, discretion is required in the interpretation of genotypic HIV DR testing data derived from samples with VL levels < 100 copies/mL when this protocol is applied. This is especially crucial when LADRVs are of special interest in the genotypic analysis of LLV specimens.

The protocol was optimized to ensure its efficiency in amplifying all examined HIV subtypes at varied VL levels, including LLV. This protocol employs a manual nucleic acid extraction approach in combination with regular PCR procedures, which is readily available in both resource-rich or resource limited countries. As it stands, the required plasma volume is only 400 *μ*L, which is achievable for most of the laboratories even in RLS. However, when resources permit, this protocol can be further customized by increasing the initial plasma input, preconcentration of HIV viruses via ultracentrifugation, or reducing the elution volume during the HIV RNA extraction stage when low VL is expected. These will help to boost the HIV template input to the initial RT-PCR and eventually increase the success rate of subsequent amplifications. This is especially beneficial when HIV-1 subtype D specimens are concerned.

## 4. Conclusions

This newly developed HIV-1 PCR protocol is highly sensitive and robust in successful amplification of HIV-1 PR and RT genes. This protocol employs inexpensive manual nucleic acid extraction and well-optimized PCR conditions, which is highly cost-effective and readily available in all biomedical laboratories. It performs consistently on HIV-1 specimens with varied subtypes and VL levels. This protocol may find application in any genotypic HIV assays that involve PCR amplification of HIV PR and RT genes especially when LLV is concerned. Notably, this method did display reduced consistency in amplification of HIV-1 subtype D when the VL is ≤1,000 copies/mL and, therefore, template enrichment procedures may be required to warrant successful PCR amplification of these specimens.

## Figures and Tables

**Table 1:** Primers used for RT-PCR and nested PCR.

	Primer name	HXB2 loci	Sequences 5′ – 3′
RT-PCR	PR_2071_F1	2071–2095	GAR AGA CAG GCT AAT TTT TTA GGG A
	RT_3370_R1	3348–3370	ATC CCT GCA TAA ATC TGA CTT GC

Nested PCR [one-amplicon]	PR_2243_F2	2243–2266	CTT TAR CTT CCC TCA RAT CAC TCT
	RT_3326_R2	3304–3326	CTT CTG TAT GTC ATT GAC AGT CC

Nested PCR [two-amplicon]	PR_F1	2243–2266	CTT TAR CTT CCC TCA RAT CAC TCT
	PR_R1	2761–2785	TCT CTG AAA TCT ACT AAT TTY CTC C
	RT_F2	2692–2713	CAA AAA TTG GGC CTG AAA ATC C
	RT_R2	3249–3270	CTG TCC ATT TRT CAG GAT GRA

*Notes*. The PR_2071_F1 primer was from Dr. Richard Harrigan's Lab (University of British Columbia, Canada).

**Table 2:** Details of control samples from EQAPOL.

EQAPOL ID	GenBank Accession #	HIV-1 subtype
DEMA108RU003.01	KF716491	A1
DEMA106ES002.01	JX140651	
DEMA111KE002.01	KF716474	

DEMB11US015.01	KC473835	B
DEMB10VE001.01	JX140659	
DEMB11US004.01	KC473832	

DEMC06ES003.01	KC473844	C
DEMC09ZA114.01	JX140669	
DEMC07BR003.01	JX140663	

DEMD11UG003.01	KF716480	D
DEMD08UG001.01	KC596072	
DEMD10UG004.01	KF716480	

**Table 3:** EQAPOL sample amplification using optimized protocol.

Subtypes	Sensitivity by VL (copies/mL)^*∗*^						
	5000	2500	1000	500	200	100	50
A1	+++	+++	+++	+++	+++	+++	+−−
B	+++	+++	+++	+++	+++	+++	−−+
C	+++	+++	+++	+++	+−−	−+−	−−−
D	+++	+++	+++	−−−	+−+	−−−	−+−

^*∗*^Each +/− sign represents an individual run of the same sample type, run in triplicate.
